# Exploring Empowerment in Group Antenatal Care: Insights from an Insider and Outsider Perspective

**DOI:** 10.3390/healthcare13151930

**Published:** 2025-08-07

**Authors:** Florence Talrich, Astrid Van Damme, Marlies Rijnders, Hilde Bastiaens, Katrien Beeckman

**Affiliations:** 1 Department of Public Health, Faculty of Medicine and Pharmacy, Vrije Universiteit Brussel (VUB)—Campus Jette, Laarbeeklaan 103, 1090 Jette, Belgium; astrid.van.damme@vub.be (A.V.D.); katrien.beeckman@uzbrussel.be (K.B.); 2 Departement of Nursing and Midwifery Research Group (NUMID), Universitair Ziekenhuis Brussel (UZB), Laarbeeklaan 101, 1090 Jette, Belgium; 3 Department of Child Health, TNO, Schipholweg 77, 2316 ZL Leiden, The Netherlands; marlies.rijnders@tno.nl; 4 Department Family Medicine and Population Health, Faculty of Medicine and Health Sciences, Universiteit Antwerpen, Prinsstraat 13, 2000 Antwerpen, Belgium; hilde.bastiaens@uantwerpen.be

**Keywords:** empowerment, group antenatal care (GANC), group-based care, pregnancy, perinatal care, antenatal care, qualitative research, patient-centered care

## Abstract

**Background:** Empowerment during pregnancy is linked to improved maternal and infant health outcomes and greater maternal well-being. Group Antenatal Care (GANC), a participant-centered model of care, promotes empowerment, active engagement, and the deconstruction of hierarchy between participants and care providers. It combines health assessment, interactive learning, and community building. While empowerment is a core concept of GANC, the ways it manifests and the elements that facilitate it remain unclear. **Method:** We conducted a generic qualitative study across four organizations in Brussels, using multiple data collection methods. This included interviews with 13 participants and 21 observations of GANC sessions, combining both the insider and outsider perspective. An adapted version of the Pregnancy-Related Empowerment Scale (PRES) guided the interviews guide and thematic analysis. **Results:** We identified seven themes that capture how empowerment occurs in GANC: peer connectedness, provider connectedness, skillful decision-making, responsibility, sense of control, taking action, and gaining voice. Several aspects of GANC contribute to empowerment, particularly the role of facilitators. **Conclusions:** This study highlights how GANC enhances empowerment during pregnancy through interpersonal, internal, and external processes. Important components within GANC that support this process include the group-based format and the interactive nature of the discussions. The presence of skillful GANC facilitators is an essential prerequisite. In a diverse and often vulnerable context like Brussels, strengthening empowerment through GANC presents challenges but is especially crucial.

## 1. Introduction

Empowering women is recognized as a critical factor in improving health outcomes across various stages of life, including pregnancy and childbirth [1]. Empowerment is defined as the process of enhancing individuals’ or groups’ capacities to critically assess their situations and take actions to improve them [2]. Empowerment during pregnancy has been linked to better maternal and infant health outcomes, including improved nutritional status, reduced rates of preterm birth and low birth weight [3,4], as well as improved use of maternal healthcare services [5]. Moreover, empowerment positively impacts a woman’s psychological well-being during the childbearing period, enhancing her preparedness to face the challenges of postnatal life and adapt to her new role as a mother [4,6].

Studies demonstrate that a positive childbirth experience is essential for pregnant individuals [7]. Placing the woman at the center of her care, as opposed to prioritizing the needs of the care provider or institution, is a key principle [8,9]. This involves actively engaging her in decision-making and ensuring she retains dignity and a sense of control within a safe and empathetic environment. These factors align closely with the core principles of empowerment [10]. In addition, it is a reciprocal relationship: a positive birth experience not only results from empowerment but also contributes to empowerment [11,12].

Given the importance of empowerment during the first 1,000 days (counting from conception) and beyond, strategies to enhance it within perinatal care should be prioritized. One approach, recommended by the World Health Organization [13] is Group Antenatal Care (GANC), a participant-centered model of care designed to foster empowerment and active engagement. GANC, of which the most well-known is the CenteringPregnancy model, integrates health assessment, social support, and interactive learning [14,15]. These components are woven into a series of ten 2 h group sessions (nine during pregnancy and one postpartum). Each group includes 8–12 participants with similar due dates, consistent healthcare providers (e.g., midwife or obstetrician), and, if desired, significant others. Participants in GANC actively engage in their care, measuring their own weight and blood pressure, while other group-members receive a brief, private physical assessment. During group discussions, healthcare providers act as facilitators rather than instructors, employing interactive methods to encourage participants to share knowledge, personal experiences, and questions on pregnancy-related topics such as breastfeeding, psychological well-being, and family planning.

Compared to conventional antenatal care, GANC achieves comparable or better maternal and neonatal outcomes [16,17,18], with participants reporting higher satisfaction [19]. Care providers report increased job satisfaction and appreciate the shift in their professional roles [20].

Internationally, various models of antenatal care exist, ranging from midwifery-led to obstetrician-led or mixed models, each offering specific advantages depending on the context in which they are implemented. In Belgium, antenatal care is mainly obstetrician-led and structured in one-to-one consultations [21,22]. Parents to be, express a need for more comprehensive care, including continuity, and richer informational support [21]. Many also value opportunities to build trust relationships with professionals and to take a more active role in their care.

Several components of GANC aim to enhance empowerment and dissolute hierarchy. These include facilitative leadership, consistent group members (same facilitators and participants throughout all sessions), and conducting interactive discussions in a circle, which promotes equity, openness, and active participation.

Previous studies investigating the impact of GANC on participants’ empowerment have produced mixed results [23,24,25]. Qualitative research that examines participants’ experiences of empowerment within GANC suggests a positive evolution as a result of participating in groups [26,27,28]. However, this topic requires further exploration across diverse contexts. While empowerment is frequently cited as a central mechanism of change within GANC, there is substantial variation in how the concept is conceptualized and operationalized [29]. This study seeks to address these gaps by investigating how empowerment is experienced and manifested in GANC settings in Brussels, by combining participants’ narratives with observational data.

## 2. Materials and Methods

### 2.1. Design

We conducted a generic qualitative research study [30], incorporating elements from multiple qualitative traditions (such as phenomenology and ethnography) rather than adhering strictly to a single framework or tradition. Owing to its flexible nature, it allows us to combine the emic (insider) and etic (outsider) perspectives. In our study, the emic perspective is represented by the participants of the GANC sessions, while the etic perspective is provided by the researcher observing those sessions.

This study is rooted in constructivism, a paradigm that recognizes how perceptions and our understanding of reality are shaped by lived experiences, cultural influences, and other factors [31,32]. The researcher’s subjectivity is viewed as an asset, acknowledging that data collection, coding, and interpretation are inherently subjective processes [33]. Subjectivity refers to the understanding that everyone brings their own perspective and beliefs, shaped by context and interactions with others.

### 2.2. Context

We conducted this study in the Brussels metropolitan region, the capital of Belgium. We approached the four organizations currently offering GANC, which, to our knowledge, are the only organizations offering GANC in Brussels. These are the Community Health Centre De Brug, Community Health Centre Medikuregem, Aquarelle ASBL, and University Hospital Brussels (UZ Brussel). These organizations primarily provide GANC to pregnant individuals in vulnerable contexts, as most GANC settings in Belgium do. These are identified based on criteria outlined in the tool developed by Amuli et al. [34] and consider indicators, such as financial situation, housing situation, social support, depression, anxiety, substance use, and domestic violence.

### 2.3. Participants

The participants were pregnant individuals attending GANC sessions at one of the participating settings. We chose convenience sampling (i.e., a non-probability sampling method that selects participants based on factors such as accessibility, availability, and willingness to participate) [35,36], given the specific and limited availability of GANC services in Brussels and the potential participants. The sample size was not predetermined; it was guided by the principle of data sufficiency: sampling continued until data provided rich, varied, and meaningful insights [37]. A total of 13 GANC participants were recruited. Eligibility criteria included participants who participated in at least five sessions and who were 36 weeks pregnant, to ensure substantial engagement with GANC sessions. Minors were excluded. The interviews were conducted by a trilingual researcher (FT) proficient in Dutch, French, and English. For participants whose native language was not one of these three, interpretation was offered. None opted to use this service. Written informed consent was obtained from participants. In addition, we observed GANC sessions in four groups. Participants were interviewed from both observed and non-observed groups.

### 2.4. Data Collection

The semi-structured interviews were conducted by FT between August 2021 and November 2022. The researcher (FT) is trained in qualitative research. She is also a trained midwife, which supports her listening skills and sensitivity when interviewing participants. In addition, she completed training in GANC. For the interviews, the participants chose the time and location (i.e., at their home or at the GANC setting). Participants were asked about their experiences with GANC, without explicitly referencing empowerment, to minimize socially desirable responses and because of the ambiguity of the concept “empowerment” [10]. Interviews began with open-ended questions, with follow-up questions guided by their responses. Questions were guided by the framework of the Pregnancy-Related Empowerment Scale (PRES) [38]. The PRES, the only available scale measuring pregnancy-related empowerment [39], evaluates four domains: provider connectedness, skillful decision-making, peer connectedness, and gaining voice (see Table 1 for definitions). The PRES framework is grounded in the concept of health-related empowerment and integrates social theory [40], feminist theory [41], and Bandura’s theory of self-efficacy [42]. The four domains of the PRES are considered manifestations of empowerment. This study sought to examine whether these domains and other elements of empowerment are present in GANC and to explore the mechanisms through which they emerge. To detect other domains beyond those of PRES, we kept the general definition of empowerment described by Portela and Santarelli in mind [2]: “an ongoing process of enabling individuals and groups (1) to improve their capacities, (2) to critically analyze situations, and (3) to take actions to improve those situations”. FT developed the interview guide, which enabled her to become familiar with the content and intent of the questions. Prior to data collection, she further familiarized herself with the PRES framework and the interview guide through repeated review and internal discussions with the research team. We used the interview guide flexibly to allow for the emergence of empowerment elements beyond those defined by the PRES and to be guided by the narrative of the interviewee.

In addition, the researcher conducted structured and unstructured participatory observations of GANC sessions of four groups across the participating organizations. These took place from February 2021 to October 2022. Participatory observation was chosen [43] to minimize disruption to group dynamics. The observations focused, as in the interviews, on the four PRES domains and the definition of Portela and Santarelli [2]. Detailed field notes were made.

While interviews revealed participants’ own meanings and interpretations, the observations add richness to the findings, allowing the researcher to capture non-verbal dynamics, social interactions, and contextual nuances that participants might overlook or find difficult to articulate. Observations and interviews were conducted concurrently and inevitably influenced one another. In line with the constructivist perspective, this data triangulation aims to explore and acknowledge multiple realities, rather than identify inconsistencies in data and uncover a single objective truth. The COVID-19 pandemic was ongoing during the study period; however, no extra safety measures were required.

### 2.5. Data Analyses

We applied thematic analysis [44,45] with a primarily inductive coding approach, developing themes from the data, while using the domains of the PRES as a guiding framework. In addition, we opted for latent coding to interpret underlying ideas, meanings, and assumptions beneath the data, thus moving further from the explicit content. Data from the observations was coded based on the codes and themes retrieved from the interviews. The analytical process was both repetitive and iterative, with insights evolving through multiple rounds. Analysis began during data collection and followed the six phases of reflexive thematic analysis outlined by Braun and Clarke [45]. FT familiarized herself with the data, transcribed interviews, and repeatedly reviewed the transcripts and observation notes. She then coded the dataset, grouped the codes into potential themes and subthemes, reviewed these themes against the coded extracts, defined and named the themes, and ultimately presented the themes alongside relevant data extracts in the report. NVivo software version 13 was utilized for data management and organization.

### 2.6. Reflexivity

The research team (FT, AVD, KB) undertook regular discussions to support reflexivity [46]. AVT familiarized and coded three transcripts. In addition, meetings with and written feedback from all co-authors provided additional perspectives. This approach was undertaken from the study’s outset to the analysis and writing. The aim of this collaborative research process was to achieve a richer interpretation, rather than a consensus of meaning [47]. The researchers and their choices aimed to be sensitive and accountable to the needs of the participants and GANC settings. Finally, combining observations and interviews was a strategy to ensure the researcher’s interpretation aligned with the participant’s perspective. FT continuously reflected on her background, assumptions, and dual role as both practitioner and researcher. Her perspective as a midwife, sociologist, and public health professional with direct GANC implementation experience offered unique insights into empowerment within GANC. Simultaneously, her academic training in qualitative research supported critical distance. Shared traits with participants, such as being a pregnant woman of similar age, helped build rapport, while differences in professional role and researcher status were acknowledged.

## 3. Results

### 3.1. General Findings

The GANC-settings varied in terms of location, organization of (prenatal) care, and patient population (Table 2). Within the four GANC settings, 21 observations and 13 interviews with participants (25 to 93 min) were conducted. One organization did not organize groups at the time when interviews were conducted.

Group sizes ranged from three to seven participants (average = 4). The series comprised six to nine sessions per organization. The groups are formed based on gestational age. Each group session lasted between 120 and 175 min in length. The groups were facilitated by the same two midwives. The spoken language was French, while a facilitator translated into English or Dutch if necessary.

We identified seven themes that describe participants’ experiences with GANC in relation to empowerment. In addition to the four elements outlined in the PRES framework (i.e., peer connectedness, provider connectedness, skillful decision-making, and gaining voice), we identified three additional themes: sense of control, responsibility, and taking action. Each theme is a manifestation of empowerment. The seven themes/domains are categorized under three processes: (1) interpersonal, (2) internal, and (3) external processes.

**Figure 1:**
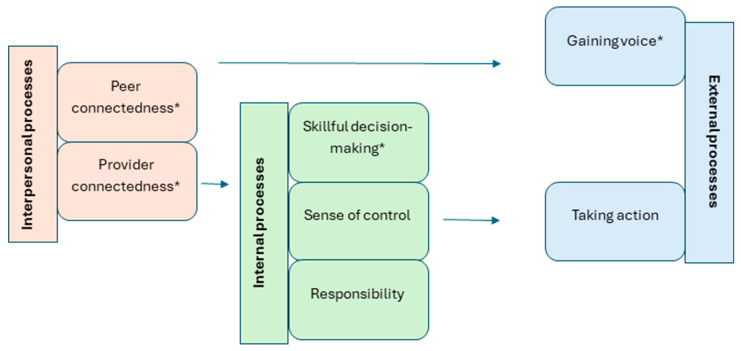
Illustration of the themes. *: domains of PRES.

Interpersonal processes refer to the connections between participants as well as the relationships between participants and care providers. Internal processes encompass the mental and emotional shifts that occur through GANC participation. These internal changes subsequently externalize and become visible through actions and interactions with others, referring to the external processes. Each process can be viewed as distinct, but at the same time interconnected, with each one enabling the next level. Figure 1 illustrates the seven themes, organized under their corresponding processes, and highlights the interconnections between these processes.

### 3.2. Interpersonal Processes

#### 3.2.1. Theme 1: Peer Connectedness

Peer connectedness, the bond between participants, was found to be a central theme in participants’ experiences of GANC and was described as profound. Interestingly, the observer did not visually notice a pronounced bond as described by participants, which suggests the possibility that this bond is something internally experienced rather than directly observable. Although interaction was minimal during informal moments (i.e., during arrival, break, and after group discussions), engagement significantly improved during group discussions. Most participants reported having little to no contact with one another outside the sessions, suggesting that the connection was limited to the context of the GANC sessions.

During interviews, participants mentioned that these profound connections are created through the sharing of experiences and learning from each other, a process facilitated by GANC’s creation of a safe space: *“We could each give our opinion on things, we were free to talk. We could say what we thought and sometimes even disagree with them (facilitators).”* (P1).

The interactive nature of the group discussions was beneficial for the connection between participants. This interaction was reciprocal: participants not only shared their own experiences but also listened to and learned from others. They valued the mutual exchange and supportive environment, as one participant described: *“It’s all about sharing. It’s great because you get to bond with the other mums too. It makes the course more human.”* (P10).

Hearing the perspectives of others was valuable to all participants. By listening to others’ stories, participants identified common ground, recognizing shared experiences and struggles. This facilitated connection and understanding despite differences in background: *“You feel reassured by the fact that they understand you, that you’re in the same situation as everyone else. And then, because everyone can talk about their problems, you say to yourself “I’ve got that too. I’m not alone.”* (P11).

At the same time, some participants noted that differences in background were enriching. These differences broadened their perspectives, opened up views, and fostered compassion, which are experiences they might not have encountered in individual follow-up care. As one participant reflected, *“I think the fact of belonging to a group, always allows us to mature (…) And then to have more compassion for others, to understand others. Because sometimes you see people, you don’t expect to hear certain stories. (…) It’s great because if we weren’t in this context, I’d never have been able to talk to these mums (with a different background). I’d never have known what they had to share.”* (P10).

The possibility of hearing diverse perspectives and comparing experiences was also beneficial to contextualize their situation. They could position themselves relative to others and put their own challenges into perspective. As one participant noted, *“Uh, learning to put things into perspective. That’s maybe the most valuable thing I took away from the sessions. That by hearing such different perspectives from such a diverse group of women, with different backgrounds, different kinds of problems, different insights, you start to see your own situation more clearly, more in perspective. Yes, sometimes it’s hard. But also, it helps you appreciate things, like: this is actually fine, or: I don’t need to worry so much.”* (P2).

In contrast, participants in groups with significant language or cultural differences often found it challenging to connect and sometimes felt excluded: *“Mmm yes it was basically saying ok Bonjour, ça va (laughs). That’s it. Yes (…) very shallow.”* (P6).

Both observations and participant interviews highlighted that, in these diverse contexts, the facilitators’ ability to enhance group cohesion, encourage active participation, and create a safe space for open sharing was crucial in building connections among its members.

In addition to the interactive group discussions and creation of a safe space, the following elements encouraged bonding between members according to the participants: introducing a break, helping each other to measure blood pressure, consistency of group members, having similar gestational ages, and in-person sessions. The following participants illustrate some of the elements: *“So that’s also nice when you see the same faces over again”* (P12), *“What’s interesting is that we’re more or less at the same due dates.” (P10), “Approachability in the sense that (…) you see each other in person. (…) You talk to someone more easily than when having to unmute yourself and to say something.”* (P2). The opposite was also observed: when, for example, a break was missing, peer interaction tended to decrease. In addition, language barriers complicated interaction for some participants.

#### 3.2.2. Theme 2: Provider Connectedness

A key feature of GANC is the development of connections and relationships between participants and facilitators. This bond is supported by continuity in both care provider and care itself, delivered over the course of six to nine sessions in the current settings. This structure allows participants and facilitators to truly get to know one another, as illustrated by one participant: *“You see each other so often. And you know each other. They know my story*.*”* (P12).

Additionally, most participants valued the availability and accessibility of the facilitators. This was appreciated both during the sessions: *“The first time I feel nervous because I don’t know the language, but she tells me, ‘No problem with the language, I will help.’”* (P5), and beyond the sessions: “*At any moment. We can reach them at any time*.” (P8).

Participants described the facilitators as understanding, respectful, kind, and patient. These qualities were critical for a positive experience, enabling participants to build a connection and feel comfortable opening up. As one participant described, *“I think that a person who is (…) welcoming, who’s a good listener, who’s at ease with you, that puts you directly at ease”* (P13). This was especially important given the period of change and vulnerability: *“Your whole life changes. t’s actually a pretty intense time. So if you have someone you can build a relationship of trust with, it becomes easier to ask what might feel like a stupid question. Or to say something like, ‘I’m worried about this, is that normal?’”* (P12).

According to participants’ experience and observations, the atmosphere during sessions was casual, familiar, and personal. The following participant describes these findings: *“It’s really like being invited into the home of someone you know and having a chat. You don’t feel like you’re at a medical appointment or something… Because they’re very friendly. Also when you arrive they ask “How are you? How did your week go?”* (P10). Observations reveal that upon entering the room, participants are personally welcomed by name, encouraged to take the time to settle in, engage in informal conversations, and enjoy a drink or snack. This relaxed and unhurried atmosphere extends throughout the sessions and during group discussions. While all participants highlighted the importance of this approach, those who did not speak the language expressed particular gratitude for the facilitators’ patience and the time they dedicated. As one participant shared, *“Always, if you want to talk, if you want to explain something, they give you the time and explain everything. They are truly wonderful.”* (P8).

The relationship between participants and facilitators undergoes a transformation in GANC, shifting from a hierarchical dynamic to one that is more collaborative and egalitarian. This is partly due to the casual atmosphere created during sessions. The physical arrangement of sitting in a circle and the absence of uniforms help to reinforce this sense of equality: *“I feel that even with the midwives, because we’re all in a circle, it doesn’t feel hierarchical or like a classroom. It feels more like we’re learning from each other.”* (P10).

Furthermore, GANC facilitators intentionally step back from their role as experts by activating participants. For example, participants are encouraged to take their own blood pressure and take the lead in group discussions. This activation of participants was described as important to become co-creators of the care process rather than passive recipients: *“There are things I can do on my own. For me, it’s important to feel useful, to feel involved in what’s happening. So (…) I think to myself: when you come to GANC, you take your own weight. It’s the least I can do. That way, at least, you see your weight for yourself. It’s not just someone else taking it and telling you.”* (P1).

In one setting, observations indicated that this activation of participants was less present. This sentiment was also reflected in a participant’s feedback and influenced her experience with GANC: *“We are not triggered, we are not, uh, switched on. Because you are actually continuously listening. And you are switched on, but in a different way. I’d prefer to be active for a while instead of passive, so to speak.”* (P6).

Interestingly, some participants regarded the facilitators as highly knowledgeable experts who guided and corrected them when needed. Their respect and appreciation for the midwives were profound, seeing them as superior to the other group members. One participant expressed her deep admiration: *“In Arabic (…) midwives are like angels, you see. (…) They take care of us and give us information that we do not know.”* (P8).

### 3.3. Internal Processes

#### 3.3.1. Theme 3: Skillful Decision Making

Skillful decision making in the context of GANC refers to the process by which participants gain the knowledge to evaluate their options and make informed choices about their pregnancy and childbirth. The process varied among participants based on their existing knowledge and preferences. For some, preferences were already partially or fully formed, while others were unaware of the available options and the decisions they would need to make. For the latter group, GANC provided new information and helped to shape their choices. Conversely, those who already had preferences found that GANC provided affirmation, offering reassurance and confidence in their choices: *“Even if you know it’s normal, it’s still important when others tell you and explain it to you. It’s more reassuring that way.”* (P9).

Several mechanisms within GANC contributed to this decision-making process.

First, participants underscored the importance of receiving objective information from the facilitators, which increased their understanding of their health and body, procedures and processes, and the range of available options: *“They (facilitators) have knowledge, they’re professionals. They can say this is scientifically proven or not.”* (P9).

Such information helped with clarity and focus in the often overwhelming information made available from various, and sometimes unreliable, sources: *“There’s just a lot of information or a lot of things you don’t know anything about. So just to have the time to learn little by little.”* (P12).Observations revealed that this was particularly evident when facilitators approached the session with a clear plan and focus. In contrast, when facilitators attempted to cover excessive detail, participants appeared overwhelmed, and interaction was limited.

Second, GANC encourages critical thinking, prompting individuals to reflect on the motivations and reasoning behind their choices. The observations revealed that self-reflection was often facilitated during group discussions through targeted questions or activities. Third, exposure to the experiences and perspectives of other participants allows individuals to evaluate their own preferences in a broader context.

Participants confirm this and perceive it as positive: *“We had to position ourselves. Uh from agree to not agree. So that you learn by doing to think about where do I position myself in relation to others. And how do others deal with that. (…) And the (facilitators) also tackled those differences to open up a discussion. (…) Why are you standing there? Why are you standing there? It’s not (…) that they want to convince (us). It was about self-reflection.”* (P2).

Variations were observed across different settings: in some contexts, critical reflection was less emphasized, with more focus placed on information and experiences.

Hearing from people who have lived and experienced childbirth/motherhood before was positively evaluated, as it was relatable and translatable to real life. As one participant noted, *“There’s the theory (…) But then there’s the reality, the experience of mums and how they’ve dealt with it on the other side. So it’s good to compare both.”* (P10).

Altogether, GANC provides a range of possibilities: *“And actually, through those testimonies and the information from the midwife, you realize oh, there’s actually a whole other spectrum of, uh, choices and ways of dealing with it. And then, based on those different insights, you figure out where you position yourself.”* (P2).

#### 3.3.2. Theme 4: Responsibility

When asked who holds responsibility for their health and well-being during pregnancy, as well as that of their unborn child, most participants identified themselves as the principal caretakers: *“Yes, you must do it in the end, right? I mean without myself, without my body there is… There is no pregnancy. You have a very big responsibility as a pregnant woman towards your unborn child.”* (P3).

The participants recognized the support and advice of others, such as healthcare providers, partners, family, and friends. However, they emphasized that the ultimate responsibility rests on their own choices and actions: *“Everyone around me is there to guide me, but in the end, I am the one in charge”* (P4).

Interestingly, the observations revealed variations in how GANC facilitators support participants to take responsibility. For instance, after group discussions about finding a midwife for postnatal care, some providers did not follow up, while others guided participants in the process or assisted them in finding a midwife. At the opposite end of the spectrum, some providers took over responsibility, arranging it on the participant’s behalf.

#### 3.3.3. Theme 5: Sense of Control

Participants reported experiencing a sense of control, highlighting their ability to influence aspects of their own health and that of their unborn child. They emphasized taking deliberate actions, such as maintaining a healthy diet and engaging in regular exercise, to support and improve their well-being.

GANC was essential in participants’ self-confidence by enhancing a sense of control and the ability to actively address: *“I feel like it’ll be fine. I have all the information I need to be able to make a decision if something comes up. (…) They (GANC) make sure you have everything you need to be able to guide the birth process yourself in the way that feels right for you.”* (P12).

Participants highlighted that the knowledge gained, both objective information and shared experiences, helped them feel more prepared for situations, even when the process, such as childbirth, remained unpredictable: *“Knowing, in general, is something that reassures me. For example, in the case of childbirth, knowing what to do if, let’s say, the baby is breech, how that is managed, or if there’s a caesarean, how that goes.”* (P11).

Additionally, participants noted that visualizing situations supported by the practical experiences of peers and the use of pictures during discussions helped them feel more prepared and equipped for what is to come: *“For example, how to deal with the first few days after the birth. It really gave me an idea of what it would be like.”* (P1).

Observations revealed differences in how settings prepared participants for childbirth. The content ranged from factual information to a focus on the emotional aspects of the event. One participant highlighted the importance of the experiential side to feel more prepared: *“So the whole medical aspect, what happens to your body has been covered. But how you experience it in your mind or the reaction that you might have. Okay, that’s different for everyone. But if you just know that it’s normal to suddenly feel frightened. Or it’s normal if at a certain moment you think it is impossible. That would be valuable to add.”* (P12).

Even though participants felt more prepared, they recognized that not everything was within their power or personal will. Through this understanding, combined with gaining a better grasp of what is within their control, the following participant experienced a greater sense of peace and reassurance: *“I feel like I know what I need to know, so to speak. And yeah, for the rest I’m kind of just going with the flow.”* (P13).

### 3.4. External Processes

#### 3.4.1. Theme 6: Take Action

The theme “taking action” captures how participants applied the acquired knowledge in practice. Through interviews, participants described how the information was functional and relevant in real-life situations. As one participant noted, *“Yes, and the group (GANC) also helped me to recognize warning signs. (…) And to know when to go to the hospital (…). To know when the pain starts, when you should go, when you shouldn’t go.”* (P3).

Participants described how their capacity to act expanded and how they made concrete efforts to improve their health, seek psychological support, and adopt new self-care practices: *“That’s why I went to talk to the psychologist a couple of times, and also why I followed the mindfulness training. You realize that you actually have a lot in your own hands. (…) Without GANC, I wouldn’t have done that.”* (P12).

Several participants attributed their behavioral changes to peer modeling. They adopted practices introduced by others in the group. One participant explained, *“I remember she (participant) said she wrote things down. Now I try to write down my feelings too. She inspired us to write as well. And everyone really listened and shared their experiences. (…) That exchange of different approaches. That was definitely something very positive.”* (P2).

Finally, self-monitoring within GANC bridges awareness and behavioral adjustment. One participant emphasized how this stimulates them to act and adjust their behavior if necessary: *“The blood pressure monitoring is interesting to do yourself. Maybe the weight a little less so, because some people can get too focused on it. But the fact that you can see, ‘ah, it’s too high,’ and you read it yourself from the monitor, it helps. When you measure it yourself, it’s easier to adjust your behaviour.”* (P2).

#### 3.4.2. Theme 7: Gaining Voice

The theme “gaining voice” reflects the freedom to express oneself within GANC sessions and beyond. Participants highlighted the opportunity to ask questions, seek clarification, and freely share their experiences, opinions, and choices. While making informed decisions is primarily an intrinsic process, GANC also facilitates the expression of these decisions openly.

Several elements contributed to this process, as described by both participants’ experience and session observations.

GANC sessions were described as unhurried, allowing participants the time and space to open up and express themselves: *“The fact that they really take the time to let everyone have their say. When you feel the need to speak or express yourself or ask questions, you feel that you’re free to do so.”* (P10). This was often not the case in other care settings, where participants often felt rushed or restricted: *“When you go to the hospital, it’s done quickly. You don’t have enough time to ask questions, to explain everything. So it’s not the same thing at all.”* (P9).

A core condition for gaining voice within GANC was the creation of a safe environment. Participants reported feeling safe to share their thoughts and experiences without fear of judgment. Participants described the group members during discussions as supportive and empathetic. As one participant noted, *“There was a very open group dynamic where no one looked down on anyone. Everyone was empathetic, at whatever you said. (…) It created an atmosphere where it was simply possible to share.”* (P12). Similarly, a participant emphasized, *“What was great was that no one felt ashamed to say how things were done (in their culture). There was no shame or fear at all.”* (P1). These experiences were confirmed by session observation, which noted that even sensitive or potentially taboo topics could be addressed openly.

Once again, the group format was beneficial, as seeing others share their experiences inspired and encouraged them to do the same: *“Uh, some already know what they want (…). That is inspiring.”* (P2).

GANC facilitators played a key role in creating the conditions for self-expression. *“They made me feel at ease”* (P13). Their use of affirming language and facilitative techniques prompted group input and validated participants’ contributions. GANC facilitators during observations would use phrases such as *“That’s an excellent question, does anyone have experience with this?”* or *“That’s a great comment, do others share this perspective?”.* One participant described how this approach reassured her: *“So I… I like it when she says yes, it’s a good question. That makes me feel more confident.”* (P4). In this way, the GANC facilitators encouraged input to emerge from the group rather than being instructed. As one participant explained, *“Because in general, they try to ask questions to see if some of us already know something about the topic, if we have answers and can explain it to others. That way, it becomes more constructive than just following a (traditional) class”* (P10).

Facilitators balanced group dynamics to ensure that all participants could share their thoughts. When necessary, they temper more dominant participants while encouraging those who are more introverted. Participants perceived an atmosphere of equality, where everyone’s input was valued, even for first-time mothers without prior experience: *“We could exchange, share our thoughts, and take ideas from others. So there were no experts, who knew more than the rest of us.”* (P1). The opposite was also true: in situations where there was less interaction due to a less facilitative style, participants perceived this negatively: *“They were mostly talking. They were continuously (emphasis) speaking. And we were continuously listening. (…) It felt more like a uh lecture so to speak.”* (P6). The same participant also noted that this inhibited self-expression: *“With her, I didn’t want to explain or express myself. I was a bit quieter around her, because I was afraid I might say something wrong.”* (P6).

Apart from the GANC facilitators’ behavior, activities and materials designed to encourage participation were described as fun, accessible, and engaging: *“The midwife shows something and asks, ‘Do you know this?’ The others who have already given birth, who have experience, immediately speak up. If they know something, they share it. So, that too, it helps.”* (P7). It creates depth in the conversations: *“We used a (emotion)dice to illustrate how we feel and let it come out that way. Because otherwise you say ‘yes, all good.’ But thanks to the animation, you reflect on it a bit more.”* (P4).

In some organizations, GANC extended its impact beyond the group setting by preparing participants to use their voice in other healthcare and social contexts. Participants reported being made aware of their rights and learning to advocate for themselves in other contexts. For instance, one participant noted feeling more confident and assertive in expressing herself, as a result of the information gained through GANC and the example of peers. *“So, during the group sessions, you really become aware of the assertiveness you, as a woman, are allowed to have. (…) It has made me more assertive in how I will stand my ground in the hospital when I give birth. And also, seeing how others handle this. Yes, it has strengthened me in my confidence to bring certain matters to the hospital”* (P2).

However, when participants faced real-life encounters outside the GANC setting, where they needed to speak up and advocate for themselves, this often proved challenging, particularly in less supportive environments. As one participant described, *“There was no time to have a conversation, or… well, I just didn’t feel like I was in the position to ask questions.”* (P12).

## 4. Discussion

Our aim was to explore how empowerment is experienced and manifested within GANC, given that empowerment is considered a central process in GANC yet remains poorly defined and understood. In contrast to previous studies that often focus on isolated aspects of empowerment, such as self-efficacy, our research examines multiple domains of empowerment. This study demonstrates that GANC offers opportunities to reshape power dynamics, thereby enabling participant empowerment. Based on our observations and interviews, we identified three key processes that underpin this transformation.

The first are interpersonal processes of connecting with peers and care providers. GANC establishes an environment that encourages openness, interaction, mutual support, and egalitarian relationships. The importance of relationships formed with both GANC facilitators and fellow participants in GANC found in our study has been well documented in previous studies [48,49,50]. Mehay et al. [29] highlights that social support, community-building, peer learning, and relational continuity are central mechanisms of GANC in general. Others have specifically linked the experience of empowerment to the emotional support, sense of safety, and relationships established within GANC [23,26,27].

In our study, the connection between group members, including GANC facilitators, is supported by visible elements, such as holding the group discussions in a circle and the continuity of group members. Even more important are the less tangible elements, such as the informal atmosphere and the extended time spent together across nine two-hour sessions. Although often anticipated as a major barrier to the implementation of GANC, our study did not identify a lack of individual time with facilitators as a challenge. This finding aligns with previous research [51].

The second kind of process towards empowerment are the internal processes. They highlight how GANC guides participants toward skillful decision-making, a sense of responsibility, and control over their own health. Central to these processes is the activation of participants in health and interactive learning, a mechanism emphasized in previous studies [29,48].

Although this is an internal and therefore individual process, our findings underscore the essential role of the group. GANC facilitators play a crucial role in determining the extent to which participants are activated. The highest level of activation occurs when facilitators encourage critical reflection and gradually transfer responsibility to participants. Additionally, the perspectives and experiences of fellow participants expand the range of options and approaches, enabling self-reflection and helping individuals to position themselves within this broader spectrum of possibilities. The study by [27] also supports our findings, demonstrating how GANC provides opportunities to explore and expand participants’ horizons. Similarly to our study, they show that diversity within the group, in terms of parity, culture, and background, can be enriching for participants.

The third process we identified are the external processes, which highlight GANC’s potential to translate what is learned in sessions into real-world, both through concrete actions and decision-making as well as through self-expression and gaining a voice. Our findings align with Wagijo et al. [52], demonstrating that GANC participants engage in healthier behaviors compared to those receiving individual care and continue these behaviors beyond pregnancy. This suggests that empowerment within GANC not only influences immediate choices but also supports sustained positive health behaviors in the long term. 

GANC is a distinct model of care that combines health assessments with interactive group-based learning. It distinguishes itself from other forms of prenatal care and education in part because of its focus on empowerment, whereas traditional approaches often prioritize knowledge acquisition as an end goal. While gaining knowledge during pregnancy is an important outcome in itself [53], GANC goes beyond the transfer of information. It encourages participants to use this knowledge as a tool to critically reflect on their choices (internal process) and to confidently articulate these choices (external process).

Another defining feature of GANC is the emphasis on peer learning. While group-based education may also occur within traditional care, in GANC, the group setting is a tool for empowerment. Peer experiences are key in this process, as participants benefit from hearing different perspectives, personal stories, and coping strategies. For example, they not only acquire theoretical knowledge about the stages of labor and birth, but also draw inspiration from others’ experiences to reflect on their own preferences regarding pain management (an internal process) and develop the confidence to express and advocate for these preferences (an external process).

As our results demonstrate, a common pitfall for GANC facilitators is reverting to a didactic style rather than encouraging peer exchange. To ensure a facilitative style and other essential elements of GANC are upheld, ongoing training, regular reflection, and peer supervision are crucial [54,55]. All facilitators in Belgium and in many other countries are trained by certified trainers from Group Care Global [56]. Although training is a prerequisite, it is important to look beyond training and explore the underlying factors that may inhibit facilitators from applying a facilitative approach in practice. This issue will be explored further in a separate study.

Lasting empowerment necessitates the involvement of all family members, including partners. The presence of partners in GANC sessions is a well-documented challenge in its implementation [57]. While some argue that a women-focused group encourages openness and a sense of safety, the downside is that it limits partner engagement in the process and perpetuates gender inequality [58].

Although this study was conducted in Brussels, its findings are relevant for both high- and low-income urban settings implementing GANC, characterized by cultural, linguistic, and socio-economic diversity [56,59]. Such diversity influences group composition, and, as our results suggest, language barriers can sometimes hinder the formation of connections between participants. The use of interpreting services may be essential to ensure that all participants, including those who do not speak the main language, can benefit from the empowering aspects of GANC. A study in the United Kingdom, conducted in a similarly diverse urban context, found that when interpreting services were provided, both learning and community support were enhanced. Importantly, the relevance of our findings extends beyond urban contexts. Across the globe, healthcare systems remain largely hierarchical, with an imbalance of power between healthcare providers and patients. GANC represents a fundamental shift in care philosophy toward a more egalitarian participatory model of care.

Building on the previous point, although GANC lays a strong foundation for empowerment, our findings suggest that participants may still struggle to feel empowered in more challenging circumstances. It is crucial to recognize that empowerment in pregnancy is not solely an individual attribute to be acquired or enhanced; rather, it is shaped and often constrained by broader political, social, and cultural structures [10,39]. Empowerment cannot be fully achieved if the external environment continues to reproduce structural inequalities.

Sustaining and strengthening empowerment beyond pregnancy is essential. Extending group-based care into the postnatal period, up to two years postnatal, could maximize GANC’s benefits by offering parents continued support, practical skills, and tailored knowledge [60]. Gresh et al. [60] advocate for an adaptation of the model that balances maternal and infant care, rather than prioritizing the latter, which is common practice. This adaptation is indeed a valuable step toward enhancing women’s empowerment during this transformative and vulnerable phase.

### Limitations and Strengths

The findings of this study must be interpreted considering the methodological choices made. Participants were recruited from both observed and non-observed GANC groups. Due to the frequent contact between researchers and participants in observed groups, a different type of relationship may have developed compared to those in non-observed groups. For example, one participant from a non-observed group appeared slightly hesitant to speak at the beginning of the interview due to the unfamiliar researcher. However, this diminished over time, and the participant later expressed that she felt at ease speaking with the researcher. Potential bias was mitigated by the use of a framework. Additionally, observations and interviews were conducted simultaneously, which may have influenced how the researcher approached the data collection and interpretation. Once again, the framework provided a consistent lens to interpret the findings. This approach also had advantages, allowing participants to elaborate on observed interactions. Furthermore, part of the study was conducted during the COVID-19 pandemic, which influenced the organization of groups, including group size, and may have impacted the findings. Lastly, the number of participants was not predetermined before data collection, as is common in qualitative research. Instead, we continued interviews until data sufficiency was achieved: recurring themes emerged, reflected through a variety of perspectives. This leads to a discussion of the transferability of our findings. The results should be interpreted within the specific context. In line with constructivism, the researchers’ aim is to contribute to the understanding of a complex phenomenon rather than explain an absolute truth, and we acknowledge both the strengths and weaknesses of this approach. However, by incorporating diverse settings and using a framework, our approach enhances transferability for both researchers and practitioners.

This study has several other strengths. First, it provides an in-depth exploration of a complex topic, offering a significant contribution to the existing literature. Second, the combination of both data collection methods enriches the results. Third, by going beyond a traditional survey approach, it ensures the inclusion of harder-to-reach individuals and those in vulnerable contexts.

Future research could explore how the participants’ inner circles, particularly partners, can be more actively involved in GANC. In addition, our findings highlight the difficulty some facilitators experience in adopting and sustaining a facilitative style. Future studies should therefore investigate the underlying reasons behind this challenge. To further examine the empowerment potential of GANC, it would be valuable to study empowerment in different contexts, using the same in-depth qualitative approach with attention to multiple domains of empowerment.

## 5. Conclusions

In conclusion, GANC promotes empowerment by focusing on interpersonal, internal and external processes. Important components within GANC that support this process include the group-based format and the interactive nature of the discussions. However, the presence of skillful and committed GANC facilitators is an essential prerequisite for its success. In a diverse and often vulnerable context like Brussels, strengthening empowerment through GANC presents challenges but is especially crucial. Expanding the model to other domains, such as postnatal care, is crucial to sustain and strengthen empowerment beyond pregnancy.

## Figures and Tables

**Table 1:** Definitions of the four dimensions of PRES [38].

Provider connectedness: A healthcare relationship that minimizes the power differential between clients and providers. These relationships are created within an environment of respect and trust. Skillful decision-making: A process by which women come to evaluate and choose a direction that will impact their health. Peer connectedness: A bond between women that develops from the evolution of caring and supportive relationships. Gaining voice: The ability of women to be knowledgeable about their health and advocate for their healthcare options for themselves and their families.

**Table 2:** Overview of the settings and participants.

	Setting 1	Setting 2	Setting 3	Setting 4	Total/Average
Type of organization (Primary/secondary care)	Community Health Center (Primary)	Community Health Center (Primary)	University hospital (Secondary)	Non-profit organization (attached to a hospital)	n/a
Regular prenatal care performed by	Midwives	Midwives	Mainly gynecologists	Midwives	n/a
GANC performed by	2 midwives	2 midwives	2 midwives or 1 midwife and 1 psychologist	2 midwives	n/a
Number of months/years of GANC experience at the beginning of observation	3 years	3 years	3 months	1 year	n/a
Target group for GANC	All pregnant people, but especially residents	All pregnant people, but especially residents	People facing psychosocial and/or socioeconomic challenges	Pregnant people with no social security and living in precarious conditions	n/a
Number of groups observed	1	1	1	1	4
Number of sessions observed	5	5	6	5	21
# women interviewed	2	2	9	0	13
# interviewed and participants in an observed group	0	2	3	n/a	5
Primiparous/multiparous	1/1	2/0	5/4	n/a	8/5
Mean age (range)	28 (24–32)	31 (27–35)	30 (21–34)	n/a	29.7
# non-native Dutch or French speakers	1	0	4	n/a	5

## Data Availability

Data is unavailable due to privacy. The authors do not have permission from the participants to share data.

## Abbreviations

The following abbreviations are used in this manuscript:

GANC	Group Antenatal Care
PRES	Pregnancy-Related Empowerment Scale
WHO	World Health Organization
